# Quantifying Intratumoral Heterogeneity and Immunoarchitecture Generated In-Silico by a Spatial Quantitative Systems Pharmacology Model

**DOI:** 10.3390/cancers15102750

**Published:** 2023-05-13

**Authors:** Mehdi Nikfar, Haoyang Mi, Chang Gong, Holly Kimko, Aleksander S. Popel

**Affiliations:** 1 Department of Biomedical Engineering, School of Medicine, Johns Hopkins University, Baltimore, MD 21205, USA; 2 Clinical Pharmacology & Quantitative Pharmacology, AstraZeneca, Waltham, MA 02451, USA; 3 Clinical Pharmacology & Quantitative Pharmacology, AstraZeneca, Gaithersburg, MD 20878, USA; 4 Sidney Kimmel Comprehensive Cancer Center, Department of Oncology, Johns Hopkins University, Baltimore, MD 21231, USA

**Keywords:** intratumoral heterogeneity, computational digital pathology, quantitative systems pharmacology (QSP), agent-based Model (ABM), immunoarchitecture, immune checkpoint inhibitor

## Abstract

**Simple Summary:**

We introduced a novel approach to quantitatively validate the performance of a hybrid spatio-temporal method called spatial quantitative systems pharmacology (spQSP). This platform is composed of a compartmental QSP model describing tumor growth dynamics, anti-tumor immune response and immune checkpoint therapy in a whole-patient and a spatial agent-based model, describing the tumor to simulate the effect of anti-PD-1 therapy (an immune checkpoint inhibitor) on simulated intratumoral heterogeneity. Four spatial metrics adopted from computational digital pathology, along with the ratio of cancer cells to immune cells, were used to categorize the tumor microenvironment as “cold”, “mixed” and “compartmentalized” patterns, which were related to the efficacy of the treatment. This study compared the intratumoral heterogeneity description capability of the metrics to facilitate future comprehensive and tangible research on specific cancer types, different therapeutics as single agents or combination therapies, and immunopathological multiplexed samples. Having a better quantitative understanding of intratumoral heterogeneity using numerical simulations can help design more effective treatments.

**Abstract:**

Spatial heterogeneity is a hallmark of cancer. Tumor heterogeneity can vary with time and location. The tumor microenvironment (TME) encompasses various cell types and their interactions that impart response to therapies. Therefore, a quantitative evaluation of tumor heterogeneity is crucial for the development of effective treatments. Different approaches, such as multiregional sequencing, spatial transcriptomics, analysis of autopsy samples, and longitudinal analysis of biopsy samples, can be used to analyze the intratumoral heterogeneity (ITH) and temporal evolution and to reveal the mechanisms of therapeutic response. However, because of the limitations of these data and the uncertainty associated with the time points of sample collection, having a complete understanding of intratumoral heterogeneity role is challenging. Here, we used a hybrid model that integrates a whole-patient compartmental quantitative-systems-pharmacology (QSP) model with a spatial agent-based model (ABM) describing the TME; we applied four spatial metrics to quantify model-simulated intratumoral heterogeneity and classified the TME immunoarchitecture for representative cases of effective and ineffective anti-PD-1 therapy. The four metrics, adopted from computational digital pathology, included mixing score, average neighbor frequency, Shannon’s entropy and area under the curve (AUC) of the G-cross function. A fifth non-spatial metric was used to supplement the analysis, which was the ratio of the number of cancer cells to immune cells. These metrics were utilized to classify the TME as “cold”, “compartmentalized” and “mixed”, which were related to treatment efficacy. The trends in these metrics for effective and ineffective treatments are in qualitative agreement with the clinical literature, indicating that compartmentalized immunoarchitecture is likely to result in more efficacious treatment outcomes.

## 1. Introduction

The interactions among cancer cells; stromal cells, such as fibroblasts and adipocytes; immune-inflammatory cells, such as T cells, B cells and macrophages; and blood and lymphatic vascular networks govern the heterogeneity of the tumor microenvironment (TME) [1]. At the population level, tumor heterogeneity can be classified as intertumoral and intratumoral heterogeneity [1,2]. Intertumoral heterogeneity is defined as differences between tumors, while intratumoral heterogeneity is related to the spatial and temporal distribution of various cell types within a tumor [1,2,3,4,5]. The results of a plethora of studies on different cancer types indicate that intratumoral heterogeneity influences cancer progression and can contribute to drug resistance [1,3,6,7]. Therefore, a comprehensive understanding of intratumoral heterogeneity can be used to inform the design of more effective treatments. 

Spatial biomarkers obtained from immunohistochemistry (IHC) and immunofluorescence (IF) images, especially multiplexed, have demonstrated great potential in predicting prognosis, tumor recurrence and response to therapy by resorting to statistical analysis and recently, machine-learning methods [4,5,8,9,10,11,12,13,14]. 

Numerical and mathematical simulations of various biological systems at different time and length scales are now common tools to inform pre-clinical studies and clinical trials [15,16,17,18,19,20,21,22,23,24,25,26,27]. In the field of cancer immunology, quantitative systems pharmacology (QSP) has been successfully utilized to model the dynamics in the whole body as a complex system of internal and external processes resulting from both disorder and treatment. However, QSP models, which are generally comprised of sets of ordinary differential equations (ODEs), cannot capture spatial intratumoral heterogeneity [15,18,19]. In contrast to ODE-based QSP, spatio-temporal agent-based models (ABM) or hybrid models can simulate intratumoral heterogeneity [15,18,19,28,29,30]. In agent-based models (ABMs), entities, or agents, interact with each other based on a set of stochastic rules that are typically informed by experimental data. Recently, spatial QSP (spQSP) has been introduced to alleviate the limitations of both QSP and ABMs to model intratumoral heterogeneity [15,18,19]. Spatial QSP (spQSP) comprises the underlying physics and parameters of ABMs, while also incorporating whole-patient dynamics, including pharmacokinetics [15,18,19]. spQSP has been used to model the TME in different cancer types and the results have been validated qualitatively. However, quantitative validation for this platform is limited [15,18,19]. It should be noted that the validation of spatio-temporal methods is generally challenging [6,31]. 

Computational digital pathology refers to the application of computer-based methods and tools for the analysis and interpretation of digitized images of pathological samples. It involves the integration of various computational techniques, such as image processing, machine learning and data mining, to extract meaningful information from digital pathology images. Recent studies on computational digital pathology [3,12,32,33,34,35,36] have introduced metrics to characterize spatial heterogeneity of the TME, specifically its immunoarchitecture. In this study, we use four spatial metrics to quantify intratumoral heterogeneity of the results obtained in spQSP simulations. Anti-PD-1 therapy is considered as an immune checkpoint inhibitor to diminish tumor growth and unleash the capability of the immune system. In some studies, tumor immune microenvironments are characterized as “hot” or “cold” in terms of their immune content [19,32]. We also categorize the TME immunoarchitecture as “cold”, “mixed” and “compartmentalized” and link these patterns to the efficacy of the treatment.

## 2. Materials and Methods

**Figure 1:**
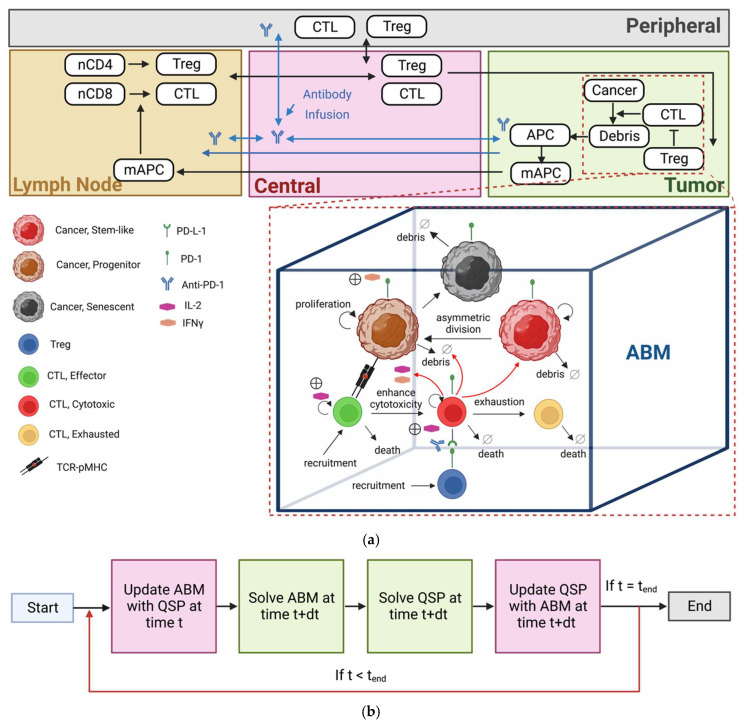
(**a**) spQSP platform schematic. Cellular and molecular interactions in different compartments. Top: QSP model: tumor-draining lymph node (LN) compartment, central (blood) compartment, tumor compartment, peripheral compartment. Mature antigen-presenting cells process tumor antigen in the tumor compartment and these cells are transported via lymphatic vessels to the lymph node where they prime naïve cytotoxic T lymphocytes (CTL) and T regulatory cells (Treg). These processes result in clonal expansion of the T cells, and these cells are trafficked through blood circulation and extravasate into the tumor microenvironment. Middle: spatio-temporal ABM module substitutes a portion of the tumor compartment from the QSP model, where different subtypes of cancer cells, including cancer stem-like cells, cancer progenitor cells, cancer senescent cells and different T cells, including CTL effector, CTL cytotoxic and CTL exhausted, are represented spatially at a single-cell resolution. Spatio-temporal distribution of soluble cytokines IL-2 and IFNγ are described by partial differential equations (PDEs). (**b**) Algorithm flowchart, coupling between the QSP and ABM modules.

Figure 1a illustrates the spQSP immuno-oncology platform comprising two modules: whole-patient compartmental QSP module and a three-dimensional spatial ABM. The QSP module comprises four compartments: tumor and peripheral compartments; tumor-draining lymph node (LN); and central (blood) compartment. In the LN, T cells are educated by recognizing tumor-specific antigens. The central (blood) compartment is responsible for transporting naïve and effector T cells to other compartments. In the tumor compartment, cancer cells interact with various immune cells (effector T cells, regulatory T cells), leading to the killing of cancer cells. Other organs and tissues are lumped into the peripheral compartment. The QSP module has been used to model non-small-cell lung cancer (NSCLC) [21] and, with some modifications, was coupled with an ABM for modeling the effect of anti-PD-1 therapy in the TME [15]. The ABM is utilized to simulate the dynamics of a portion of the tumor compartment to capture intratumoral heterogeneity over time. Due to the computational cost of ABMs, when carried out for a virtual population over the period of months, simulation of the whole tumor compartment is not feasible beyond a certain cell number, typically in the millions [15]. The ABM module is itself divided into a discrete layer and a continuum layer.

In the discrete layer, cancer cells and immune cells in different states interact (“socialize”) with each other based on the rules established in cancer immunology [37]. Figure 1a depicts cancer cells, CD8+ T cells and regulatory T cells. The rules for cancer cell differentiation are based on models developed by Norton et al. [20,38]. Cancer cells are classified as cancer stem-like cells (CSCs), progenitor cells or senescent cells. Regulatory T cells (Treg) contribute by suppressing CD8+ T cells. CD8+ T cells and Treg cells are recruited from the same entry points simulating tumor vasculature that are randomly distributed inside the voxels that have cancer cells in their Moore neighboring voxels (Moore neighborhood is defined as the cubic neighborhood of 26 voxels surrounding the current voxel). The probabilities and rules for events associated with the ABM are derived from ODEs in the QSP modules. The derivation process is described in detail in [15,18,19]. 

In the continuum layer, the distribution of two cytokines, namely Interleukin-2 (IL-2) and Interferon-Gamma (IFNγ), resulting from killing cancer cells are modeled via partial differential equations (PDEs). The finite volume method (FVM) is used to discretize and solve these equations [15,18,19,39]. For the coupling between QSP and the ABM, the probabilities of different events in the ABM are calculated based on QSP parameter values or variable values at the same time point in the simulation. The algorithm flowchart is shown in Figure 1b. The numerical procedure is initiated by solving the QSP module, and the ABM probabilities are computed based on the QSP parameters. After solving the ABM, the QSP module is solved at the next time step, and it is updated with the ABM consecutively [15,19]. The spQSP platform was developed in C++ and Python was used to plot the results. The results were visualized using ParaView software package [40]. In this study, we used the model [15] parameterized for NSCLC; however, the results and comparisons are meant to be conceptual pan-cancer, aimed at the question of how to use various spatial metrics to characterize intratumoral heterogeneity. 

**Figure 2:**
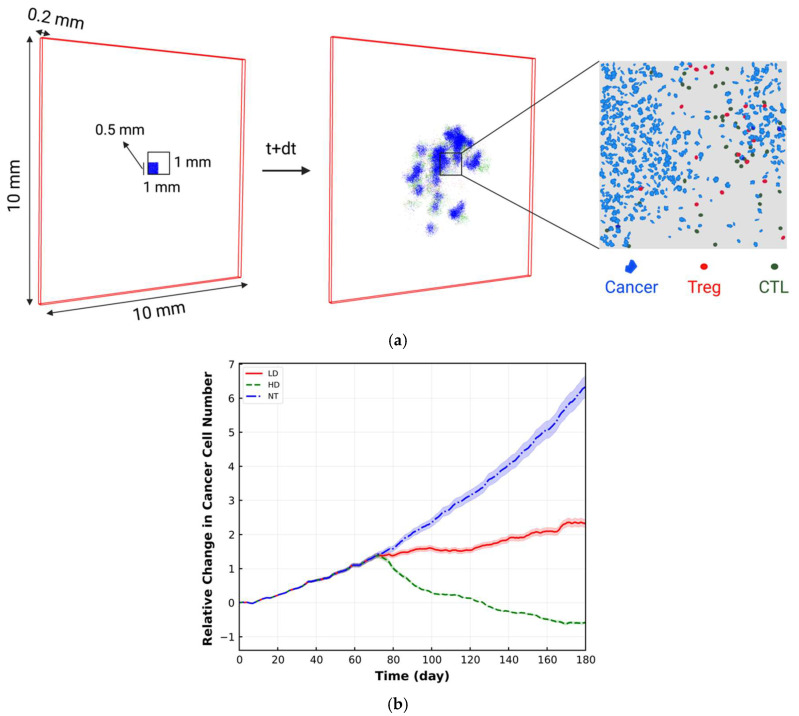
(**a**) spQSP initial condition within a flattened computational box with dimensions of 10 × 10 × 0.2 mm. All the cancer cell types, all the TCL subtypes and Treg are displayed with blue, dark green and red, respectively. The initial cancer cells are assigned to 25 × 25 × 4 voxels (0.5 × 0.5 × 0.16 mm) containing 10% CSC and 90% cancer progenitor cells. There is no immune cell in the computational box at the beginning. To make results visually comparable with multiplex immunohistochemistry and immunofluorescence, 2-D snapshots using a slice of the 3-D simulation are chosen. At each time step, different metrics are calculated on a fixed center-cropped tile from the 2-D slides; (**b**) Relative change in the number of cancer cells for high dose (HD), low dose (LD) and no treatment (NT) cases within the whole tumor. For each case, the line with different patterns is the mean of 10 replications, and the shaded band displays the standard deviation.

**Figure 3:**
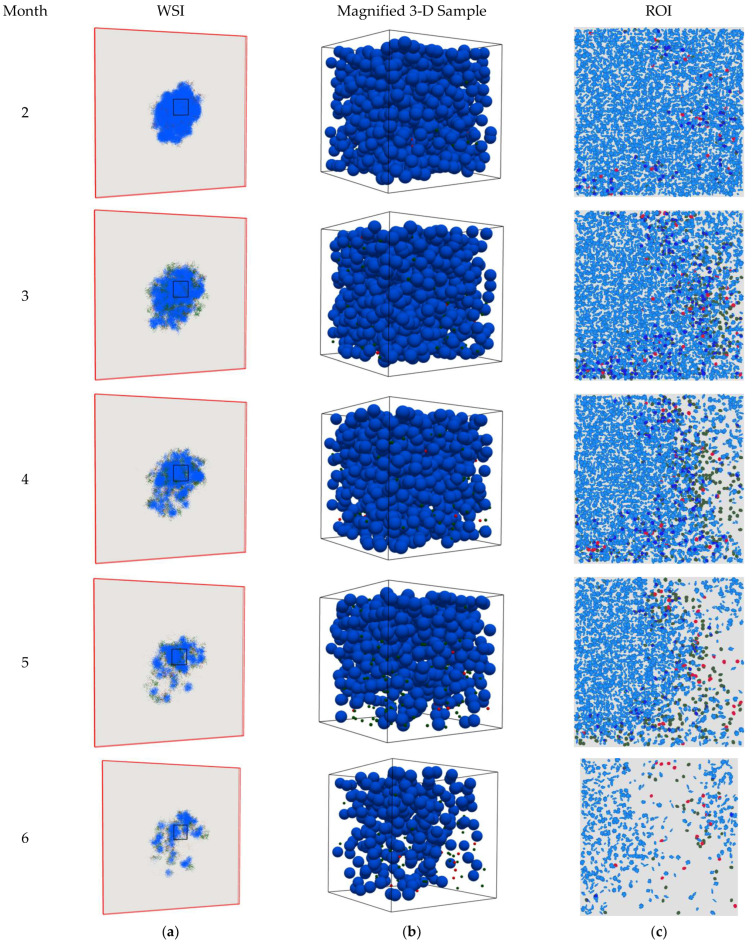
Snapshots of the spQSP simulation at different time points for the HD case. Blue: cancer cells; green: CD8+ T cells; red: regulatory T cells. (**a**) Whole slide image (WSI) represents the cell distribution on a 2-D slide in the middle of the computational box. As time progresses, the T cells penetrate into the tumor space between the cancer cells and eradicate them; (**b**) 3-D visualization in a sample cube with dimensions of 200 × 200 × 200 μm located in the center of the computation domain; (**c**) region of interest (ROI) marked in WSI represents the cell distribution on the 2-D center-cropped tile from WSI with dimensions of 1 × 1 mm. As time proceeds, TME immunoarchitecture from cold becomes mixed and then compartmentalized.

**Figure 4:**
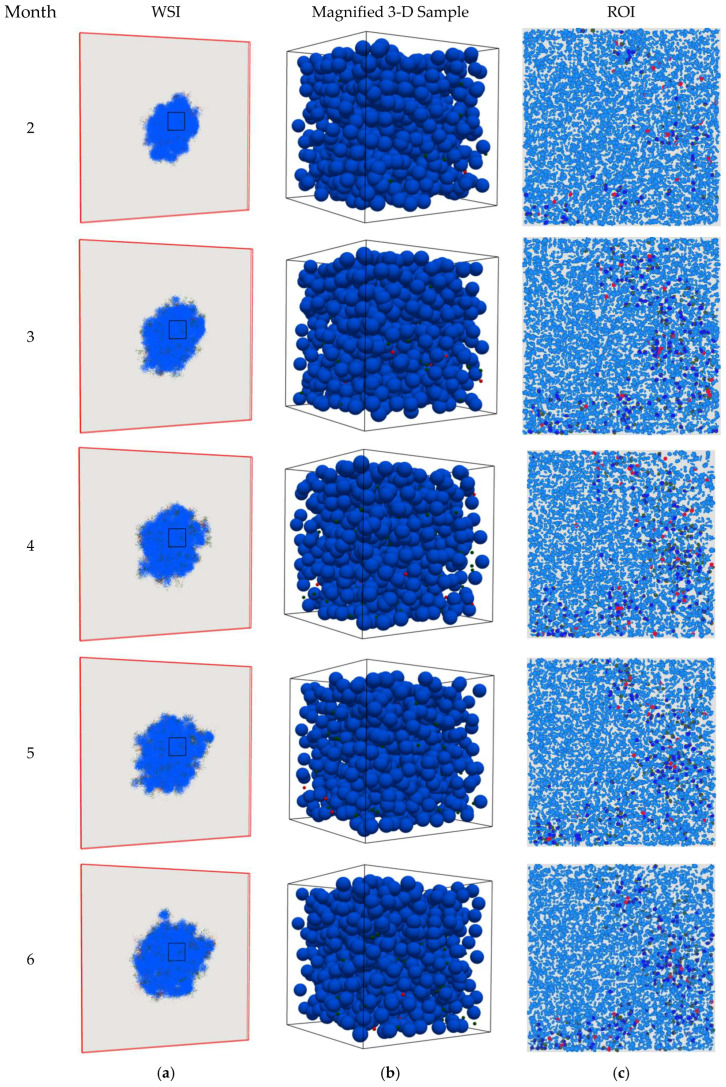
Snapshots of the spQSP simulation at different time points for the LD case. Blue: cancer cells; green: CD8+ T cells; red: regulatory T cells. (**a**) Whole slide image (WSI) represents the cell distribution on a 2-D slide in the middle of the computational box. As time proceeds, the T cells infiltrate into the tumor space between the cancer cells but cannot kill them; (**b**) 3-D visualization in a sample cube with dimensions of 200 × 200 × 200 μm located in the center of the computation domain; (**c**) region of interest (ROI) marked in WSI represents the cell distribution on the 2-D center-cropped tile from the WSI with dimensions of 1 × 1 mm. As time proceeds, TME immunoarchitecture from cold becomes and remains mixed.

**Figure 5:**
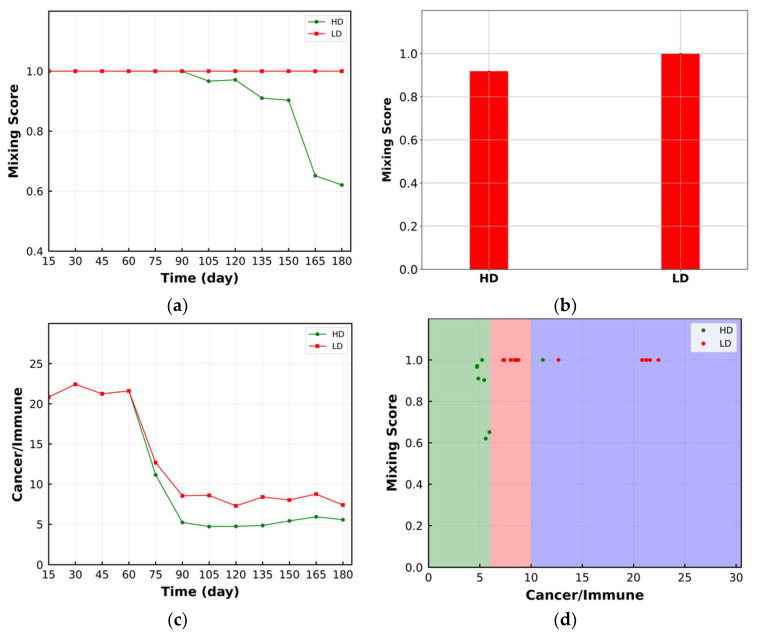
(**a**) Change in mixing score over time within the ROI. Mixing score for LD remains constant while it decreases for HD; (**b**) time-averaged value of mixing score in the format of a bar plot. LD has a higher time-averaged mixing score; (**c**) the ratio of the number of cancer cells to immune cells within the ROI. The reduction in the ratio for HD is more significant; (**d**) bivariant classification TME based on mixing score and ratio of cancer cells to immune cells (green: compartmentalized; red: mixed; purple: cold). Lower mixing score corresponds to the HD case and compartmentalized immunoarchitecture.

**Figure 6:**
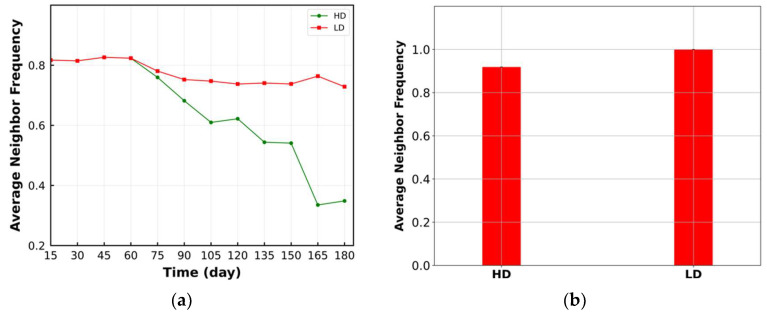

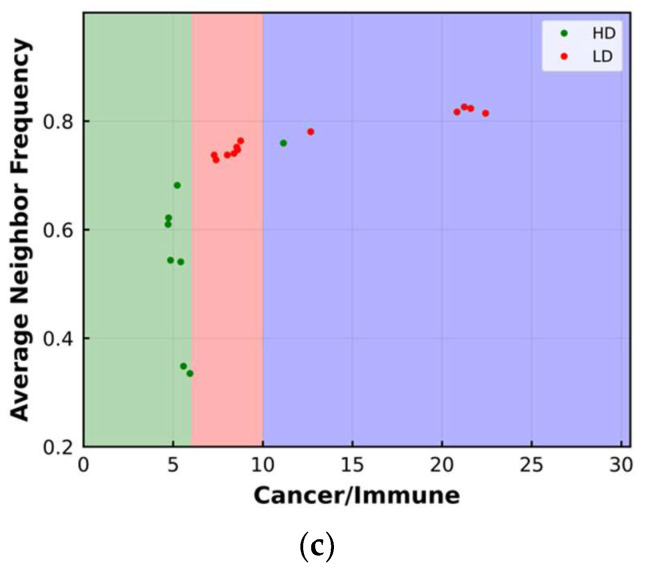
(**a**) Change in average neighbor frequency over time within the ROI. Reduction in average neighbor frequency for HD is more significant; (**b**) time-averaged value of average neighbor frequency in the format of a bar plot. LD has higher average neighbor frequency; (**c**) bivariant classification TME based on average neighbor frequency and ratio of cancer cells to immune cells (green: compartmentalized; red: mixed; purple: cold). Lower average neighbor frequency belongs to the HD case and compartmentalized immunoarchitecture.

**Figure 7:**
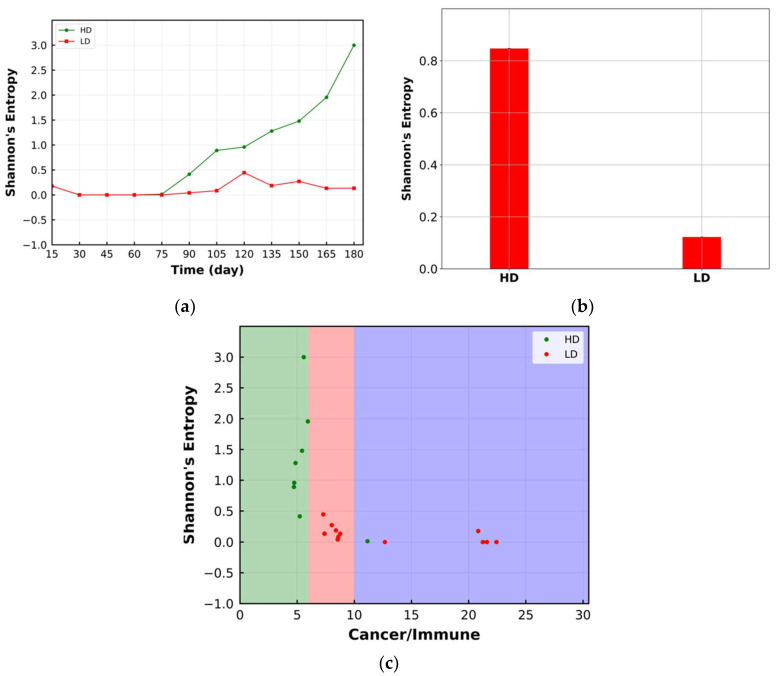
(**a**) Change in Shannon’s entropy over time within the ROI. The rise in Shannon’s entropy for HD is more significant; (**b**) time-averaged value of Shannon’s entropy in the format of a bar plot. HD has higher time-averaged value; (**c**) bivariant classification of TME based on Shannon’s entropy and ratio of cancer cells to immune cells (green: compartmentalized; red: mixed; purple: cold). Higher Shannon’s entropy corresponds to the HD case and has compartmentalized immunoarchitecture.

**Figure 8:**
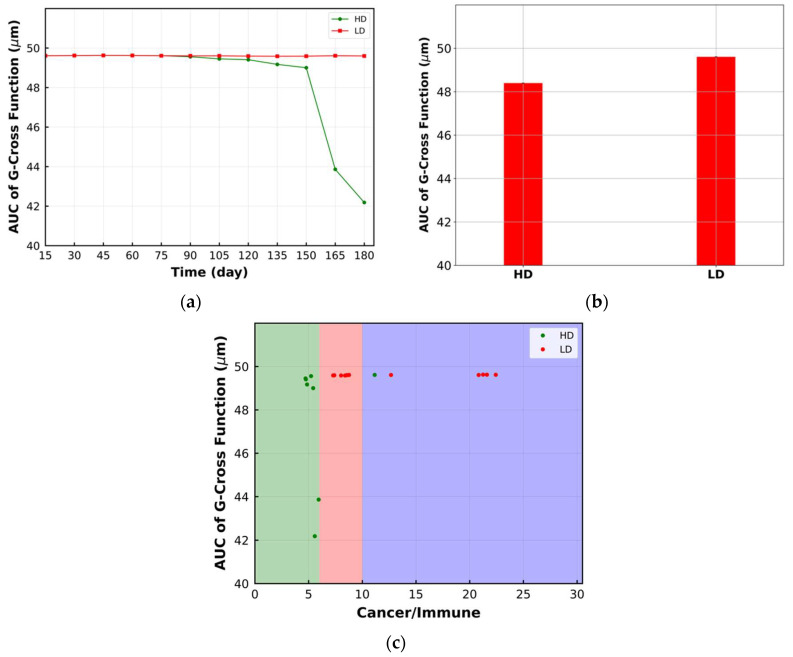
(**a**) Change in area under the curve (AUC) of G-cross function over time within the ROI. AUC of G-cross function decreases for the HD case, while it is not sensitive to the change in LD morphology; (**b**) time-averaged value of AUC of G-cross function in the format of a bar plot. LD has higher time-averaged value for the metric; (**c**) bivariant classification TME based on AUC of G-cross function and ratio of cancer cells to immune cells (green: compartmentalized; red: mixed; purple: cold). Lower AUC of G-cross function corresponds to the HD case and compartmentalized immunoarchitecture.

**Figure 9:**
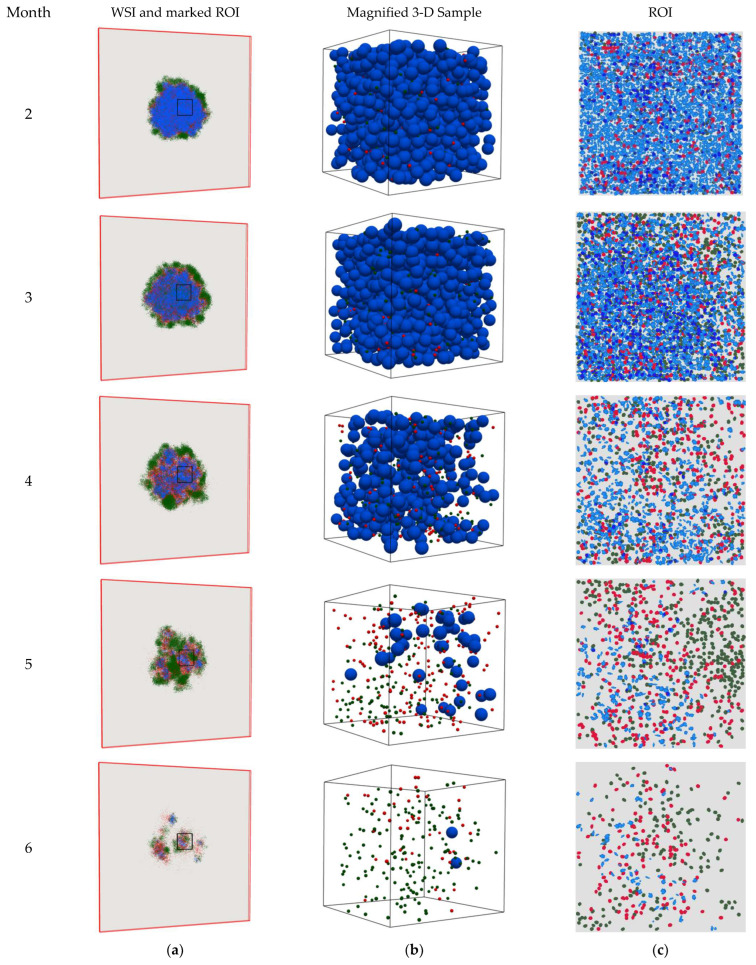
Snapshots of the spQSP simulation at different time points for an HD case with higher vascular density to extravasate immune cells (HDV). Blue: cancer cells; green: CD8+ T cells; red: regulatory T cells. (**a**) Whole slide image (WSI) represents the cell distribution on a 2-D slide in the middle of the computational box. As time proceeds, the larger number of T cells penetrates the space between cancer cells and kills them; (**b**) 3-D visualization in a sample cube with dimensions 200 × 200 × 200 μm located in the center of the computation domain; (**c**) region of interest (ROI) marked in WSI represents the cell distribution on the 2-D center-cropped tile from WSI with dimensions of 1 × 1 mm. As time proceeds, TME immunoarchitecture changes from cold to become more mixed and then compartmentalized.

**Figure 10:**
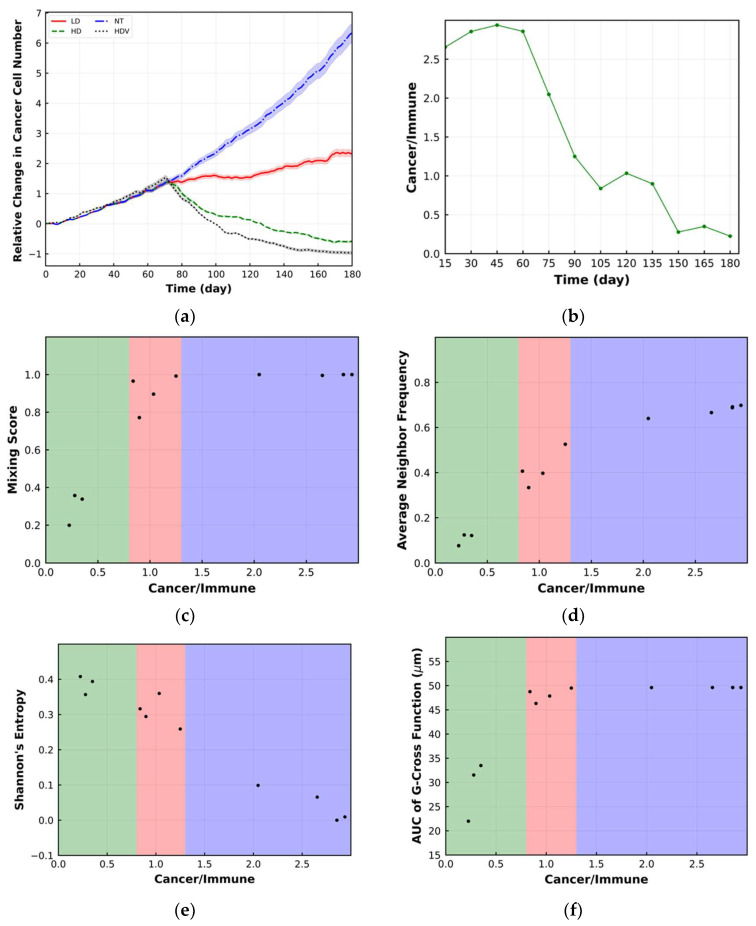
(**a**) Relative change in the number of cancer cells for HD, LD, NT and HDV with higher tumor vascular density (HDV) cases within the whole tumor. For each case, the solid line with different patterns is the mean of 10 replications, and the band displays the standard deviation; (**b**) the ratio of the number of cancer cells to immune cells within the ROI for HDV. The ratio varies between 2.6 and 0.2 over time. Bivariant classification of TME based on different spatial metrics and ratio of cancer cells to immune cells for HDV (green: compartmentalized; red: mixed; purple: cold). The following correspond to the compartmentalized immunoarchitecture: (**c**) lower mixing score; (**d**) lower average neighbor frequency; (**e**) higher Shannon’s entropy; (**f**) lower AUC of G-cross function.

**Figure 11:**
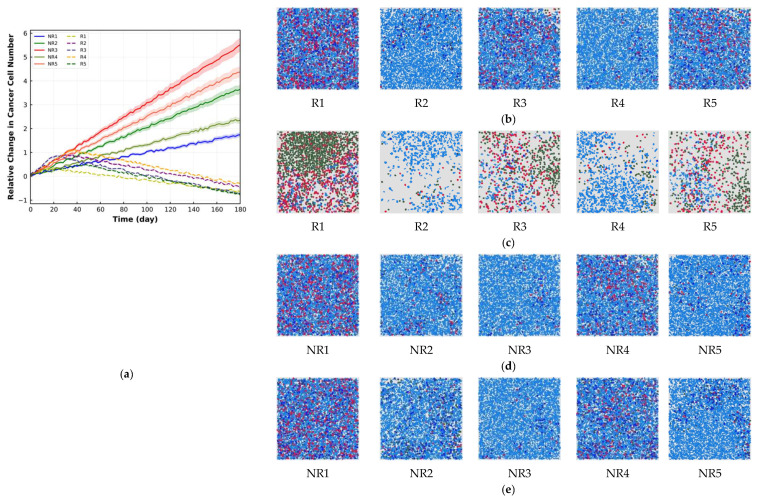
(**a**) Relative change in the total number of cancer cells for 5 responder (R) cases and 5 non-responder (NR) cases. For each case, the solid line with different patterns is the mean of 10 replications, and the band displays the standard deviation (SD). Among R cases, the final number of cancer cells is smaller in R1, R3 and R5 with respect to R2 and R4. NR3, NR5, NR2, NR4 and NR1 have the largest final number of cancer cells among NR cases. (**b**) Cell distribution in the center-cropped ROI for R cases before treatment (BT). Blue: cancer cells; green: CD8+ T cells; red: regulatory T cells. In R1, R3 and R5, the ratio of cancer cells to immune cells is smaller. (**c**) Cell distribution in the center-cropped ROI for cases after treatment (AT). R1, R3 and R5 demonstrate more obvious compartmentalized patterns because they show better response to the therapy. (**d**) Cell distribution in the center-cropped ROI for NR cases before treatment (BT). NR1 and NR4 have more mixed structure before treatment. (**e**) Cell distribution in the center-cropped ROI for NR cases after treatment (AT). The tumor-immune patterns do not change significantly for NR cases.

In this paper, we present methods aimed at the quantitative validation of the spQSP platform based on metrics derived in digital pathology. In the field of cancer immunology, quantitative validation of spatio-temporal methods is challenging and most of the methods are verified qualitatively [6,31]. Here, we use spQSP to simulate tumor progression in the presence of anti-PD-1 treatment. To make the simulations mimic the data derived from multiplexed digital pathology images, a flattened computational box is considered that is 10 × 10 × 0.2 mm in size (Figure 2a). The initial cancer cells are assigned to 25 × 25 × 4 voxels, with 10% CSC and 90% progenitor cells [15]. The size of each voxel is 20 μm and it can contain 1 cancer cell and 1 immune cell or up to 8 immune cells; thus, each domain comprises 2,500,000 voxels. There are no immune cells in the computational domain at the beginning of the simulation. To make results visually comparable with multiplex immunohistochemistry and immunofluorescence images, 2-D snapshots using a slice of the 3-D simulation are analyzed. At each time step, different metrics are calculated on a fixed 2-D center-cropped tile as the region of interest (ROI) with dimensions of 1 × 1 mm from a larger 2-D slide (10 × 10 mm) located in the middle of the computational domain as shown in Figure 2a. Because the location of the ROI does not affect the value and trend in the metrics due to a lack of modeling oxygenation and variable tumor vasculature density in this version of the model, only one ROI is used to compute the metrics. The tumor dynamics is simulated for 6 months. Two example cases are presented: a case with a high dose (HD) of anti-PD-1 therapeutic (3 mg/kg) and a case with a low dose (LD) of the same drug (0.3 mg/kg). The drug is applied at day 25. For the sake of comparison and to show the treatment effect, a case of no treatment (NT) is also simulated. Each simulation is repeated 10 times to reflect the stochastic nature of the ABM module. We calculated several spatial metrics adopted from digital pathology: mixing score; average neighbor frequency; Shannon’s (spatial) entropy; and area under the curve of the G-cross function. Results are illustrated in Figure 2, Figure 3, Figure 4, Figure 5, Figure 6, Figure 7, Figure 8, Figure 9 and Figure 10. In addition, we also simulate five responders (R) and five non-responders (NR) according to RECIST 1.1 criteria and calculate similar metrics, as shown in Figure 11. The simulations were conducted at the Maryland Advanced Research Computing Center (MARCC) using high-performance computing cluster.

## 3. Spatial Metrics

To quantify intratumoral heterogeneity, four spatial metrics are introduced, which have been used in digital pathology studies to classify tumor immunoarchitecture [3,12,19,32,33,34,35]. Three types of tumor-immune patterns have been observed in some digital pathology studies [3,12,19,32,33,34,35]: cold, with low immune cell infiltration; mixed, with high infiltration of immune cells into tumor space; and compartmentalized, in which there are regions occupied mostly by either immune or cancer cells. It has been shown that compartmentalized immunoarchitecture is commonly observed in patients with better response to treatment [32]. On the other hand, mixed immunoarchitecture is the feature associated with worse treatment results [32]. In the following subsections, we first introduce the metrics and their relations to TME immunoarchitectural patterns. We use these metrics along with the ratio of the cancer cell number to immune cell number to classify the spQSP-simulated TME in a bivariate manner [41]. The ratio of cancer cells to immune cells is also computed in the ROI. The temporal evolution of the metrics is an important capability of the model simulations that is not possible to achieve clinically or experimentally; sequential biopsies is the closest possibility, e.g., pre- and post-treatment. 

### 3.1. Mixing Score

In the application of computed patterns, mixing score is the ratio of all the immune cells within the ROI with at least one cancer cell in their neighborhood with a specific radius ranging from 30 to 50 μm. Keren et al. [32] showed that the mixed immunoarchitecture had a higher mixing score compared to the compartmentalized immunoarchitecture. In our setting, the mixing score in cold patterns is 1.0 because all the immune cells have at least one cancer cell adjacent to them. 

### 3.2. Average Neighbor Frequency

Average neighbor frequency is a localized version of mixing score in the sense that it reflects the average fraction of cancer cells in each immune cell neighborhood [34]. The relationship between this metric and TME patterns is similar to the ones for mixing score. However, its value for cold structures could be less than the corresponding mixing score. 

### 3.3. Shannon’s Entropy

Shannon’s (spatial) entropy ($E_{SP}$) is defined by $E_{SP}=-\overset{n}{\underset{i=1}{\sum }}\frac{d_{i}^{int}}{d_{i}^{ext}}p_{i}\log_{2}p_{i}$ in which $d_{i}^{int}$ indicates the average Euclidean distance between cell type *i*; $d_{i}^{ext}$ represents the average distance between cell type *i* and other cell types; and $p_{i}$ stands for the density of cell type *i* [3,33,35,42,43,44]. Here, Shannon’s entropy is computed for the cancer cells. Some findings indicate that a higher Shannon’s entropy can be linked to compartmentalized immunoarchitecture, whereas mixed TME has a lower Shannon’s entropy [35]. Shannon’s entropy for cold immunoarchitectural patterns is close to 0 as the cancer cell density (*p*) is high in the ROI. 

### 3.4. Area under the Curve of G-Cross Function

The G-cross function is defined as $G_{xy}=1-\exp\left(-p_{y}\pi r^{2}\right)$, where *x* and *y* stand for immune cells and cancer cells, respectively, and $p_{y}$ represents cancer cell density in the ROI with a radius of *r* = 50 μm, which is in the range of values used in [12]. To numerically calculate the area under the curve (AUC) of the G-cross function, $G_{xy}$ is integrated for each immune cell within the area with a radius of *r* = 50 μm. A higher AUC of the G-cross function is associated with the mixed structure and worse treatment [12]. The AUC of the G-cross function is close to the selected radius (*r*) for cold patterns [12] because the value of $G_{xy}$ reaches 1 due to the high value of $p_{y}$. 

## 4. Results

The relative change in the number of cancer cells with respect to the initial cancer cell number within the whole TME is shown in Figure 2b. For each case, the line with different patterns is the mean of 10 replications, and the band displays the standard deviation (SD). As Figure 2b illustrates, in the LD case, the rate of increase in cancer cell number is less than in the NT case, whereas in the HD case, the number of cancer cells is declining. The difference between different cases is observed approximately after day 75 because in these cases, immune cells need equal time to sufficiently infiltrate into the tumor space and start killing cancer cells. After day 75, the difference between cases becomes apparent because the dose of the drug dictates the level of PD-1 inhibition, which is related to the magnitude of cancer cell eradication by immune cells [45]. It should be mentioned that this time point depends on the base properties of cancer cells and immune cells.

The 2-D snapshots of the spQSP simulation results at different time points for the HD and LD cases are shown in Figure 3 and Figure 4, respectively. The cell distribution is portrayed in a whole slide image (WSI) on a 2-D slide and one ROI as described in Section 2. To better clarify the physics of the problem, magnified three-dimensional visualizations of cell distribution within a sample cube with dimensions of 200 × 200 × 200 μm in the center of the computational domain are also shown in Figure 3b and Figure 4b. Figure 3a,b shows that the CD8+ T cells infiltrate the tumor space and, because binding between PD-1 and PD-L-1 is inhibited by the anti-PD-1 antibody, they can eradicate the cancer cells. As a result, within the ROI, the TME immunoarchitectural patterns change from cold (before month 3) to mixed (between months 3 and 4) and end up compartmentalized (after month 4), as shown in Figure 3c. However, in the LD case (Figure 4a,b), the dose of the PD-1 inhibitor is not high enough to allow full killing action of CD8+ cells in the tumor space to lead to apparent eradication, and the TME shows mixed immunoarchitecture (Figure 4c). These observations are consistent with findings reported in the literature [3,12,19,32,33,34,35].

The change in mixing score is shown in Figure 5a. The mixing score remains constant for the LD case, and it is not sensitive enough to reflect the change in immunoarchitectural pattern from cold to mixed. For the HD case, the mixing score is reduced after 90 days, and it demonstrates the change in TME patterns to the compartmentalized mode. Figure 5b shows that the HD case has a lower time-averaged (from beginning to the end) mixing score compared to the LD case, which agrees with the literature [32]. The ratio of cancer cell number to the immune cell number within the ROI is shown in Figure 5c. In both the HD and LD cases, this ratio decreases over time, while it has a smaller value for HD after 60 days. Before day 60, the cell ratio is the same for both cases because immune cells have not sufficiently infiltrated into the tumor space. Considering Figure 5a,c, mixing score change in the ROI between the HD and LD cases starts later than the change in cell ratio, which indicates low sensitivity of the mixing score to the change in immunoarchitectural patterns. Because only the value of the spatial metrics is not sufficient to categorize TME immunoarchitecture, the ratio of cancer cells to immune cells is used as an axillary metric. Figure 5d classifies the TME to cold (purple shaded), mixed (red shaded) and compartmentalized (green shaded) patterns using the mixing score and cell ratio over time. However, the difference between HD (better treatment) and LD patterns (worse treatment) is not obvious using this type of classification. 

Figure 6a depicts the change in average neighbor frequency within the ROI over time. The trend in this metric is like the mixing score, as expected [34]. However, this metric better reflects the change in the LD TME pattern than the mixing score. Furthermore, the change in this metric between HD and LD starts as early as the cell ratio, i.e., after 60 days (Figure 5c). As Figure 6c illustrates, this metric can classify the TME for HD and LD according to the cell ratio. The HD cases have higher average neighbor frequency and compartmentalized TME (Figure 6b).

The same set of plots is shown for Shannon’s entropy within the ROI in Figure 7a–c. The HD has a higher Shannon’s entropy than LD. Considering Figure 7a, the difference in Shannon’s entropy between HD and LD is observed after 75 days. Therefore, this metric is sensitive to the change in tumor morphology [35].

Finally, the transient behavior of the AUC of the G-cross function, averaged over time and bivariant classification using this metric are shown in Figure 8a–c, respectively. This metric does not change for LD, while it shows change in TME morphology for HD with less sensitivity than previous metrics. The time-averaged value of this metric is smaller for HD, but it cannot be used to categorize the tumor morphology for HD and LD (Figure 8b,c). It should be mentioned that the difference in the metrics between the LD and HD conditions depends on the dose of the drug. As we use the dose in the LD case that approaches the HD case, the differences become less significant.

In the results presented above, our goal was to test several metrics used to characterize spatial intratumoral heterogeneity. As Figure 5c shows, in these simulations, on average, the ratio of cancer to immune cells varies approximately between 5 and 20. In the spQSP platform for specific cancer and immune cell properties, the local ratio of cancer cells and immune cells depends on tumor vascular density [3,15,35,46]. The density of tumor vasculature is also spatially heterogeneous and can be determined based on the experimental and clinical data, e.g., [47,48,49,50]. Analysis of clinical data indicates that the range of the cancer to immune cell ratios could vary widely. For example, Combes et al. [51] in a pan-cancer analysis of 12 cancer types showed that the percentage of T cells in the tumor ranges from 0 to 90%. Li et al. [52] introduced tumor purity, defined as the percentage of cancer cells in the tumor in relation to the whole tumor comprising cancer, immune, and stromal cells; tumor purity was found in the range 0.1 to nearly 1, i.e., cancer cells amounted from 10% to nearly 100% of all the cells comprising the tumor. To reflect this diversity, and to demonstrate how the intratumoral heterogeneity metrics vary in the case of low tumor purity, we increased the density of the tumor vasculature by a factor of 10 for the HD case (HDV); the results are shown in Figure 9 and Figure 10. Note that the increase in vascular density is used here as a means of increasing the immune cell infiltration; such infiltration can be achieved in vivo by a variety of immunoactivating mechanisms, including local chemokine concentrations and pharmacological interventions [53]. As Figure 9 shows, in this case, the density of immune cells is higher than the previous cases. Therefore, the tumor diminishes more than other cases as a result of immunotherapy (Figure 10a). The change in TME pattern can be observed more obviously compared to the previous cases. In this case, the ratio of cancer to immune cells varies between 2.5 and 0.5 (Figure 10b). As Figure 10 depicts, the bivariate classification using any intratumoral heterogeneity metrics and the ratio of cancer cells to immune cells can still be utilized to characterize the TME immunoarchitectural patterns with higher percentage of immune cells. The metric trends are qualitatively similar to the previous cases; however, the values of the metrics depend on the ratio of the cancer cells to immune cells. Among the metrics, Shannon’s entropy values depend on the cancer cell to immune cell ratio more strongly than other metrics (Figure 7c and Figure 10e). Here, the LD case is not shown because the ratio of cancer cells to immune cells does not change significantly, and the TME remains mixed with little change in the metrics.

**Figure 12:**
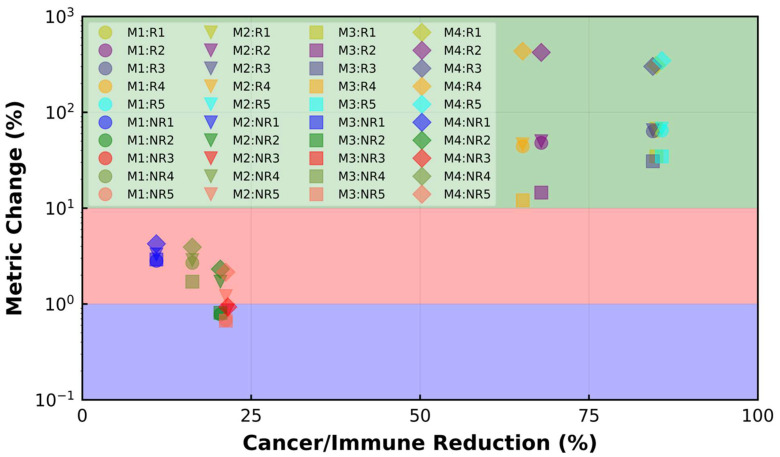
The change percentage in different spatial metrics for R and NR cases versus reduction percent of the ratio of cancer cells to immune cells. M1: mixing score; M2: average neighbor frequency; M3: AUC of the G-cross function; M4: Shannon’s entropy. Green: compartmentalized; red: mixed; purple: cold. A change of less than 1% happens when the TME remains cold during treatment. The change between 1% and 10% is linked to the mixed TME, while a change above 10% in the spatial metrics happens when the compartmentalized patterns are observed after treatment. All the R cases have compartmentalized patterns, while all the NR cases have either cold or mixed immunoarchitectural patterns.

To further illustrate the spQSP platform performance using the introduced metrics and understand each metric’s capability, we generate 10 different virtual patients, including 5 responder (R) cases and 5 non-responder (NR) cases, while the drug dose is kept constant (3 mg/kg). Each test case is simulated 10 times to reflect the stochastic nature of the ABM module. The relative changes in the cancer cell number for these cases are plotted in Figure 11a. The number of cancer cells decreases for R cases (dashed lines) and increases for NR cases (solid lines), resembling spider plots for clinical observations. For each case, the dashed lines or solid lines with different colors are the mean of 10 replications, and the band displays the SD. Among the R cases, the final number of cancer cells is smaller in R1, R3 and R5 compared to R2 and R4. NR3, NR5, NR2, NR4 and NR1 have the largest final number of cancer cells among NR cases. Cell distribution within the center-cropped ROI for both R and NR cases before treatment (BT) or pre-treatment and after treatment (AT) or post-treatment are also shown in Figure 11b–e. Among the R cases, R2 and R4 have a higher cancer cell to immune cell ratio with cold immunoarchitecture BT (Figure 11b). R1, R3 and R5 demonstrate more obvious compartmentalized patterns (Figure 11c) and they show better response to the therapy (Figure 11a). NR1 and NR4 have more mixed structures BT and AT. The NR cases’ TME remains nearly the same regardless of their cell ratio. As can be observed, R cases have compartmentalized TME patterns after treatment (AT) regardless of their TME patterns before treatment (BT), while the NR cases can have either mixed or cold TME patterns AT, depending on their TME patterns BT. The ratio of cancer cells to immune cells and the change percentage for different spatial metrics for R cases and NR cases are listed in Table 1. The change percentage of spatial metrics in R cases are higher than those in NR cases. Mixing score, average neighbor frequency and the AUC of the G-cross change for R cases are higher when the ratio of cancer cells to immune cells is lower BT. The magnitude of change in Shannon’s entropy for the dependence of the cancer cell to immune cell ratio on R cases is greater than that of other metrics. To link the change in the metrics to the immunoarchitectural patterns in TME for R and NR cases, the change percentage of different metrics versus reduction percentage of the cancer cell to immune cell ratio is plotted in Figure 12. Because the cancer cell to immune cell ratio and absolute value of the metrics vary considerably between R and NR cases, it is not feasible to categorize observed TME patterns for all the cases based on the absolute value of spatial metrics and the ratio of cancer cells to immune cells. In Figure 12, different R and NR cases are shown with different symbols and colors. In Figure 12, the change percentage in the metrics during treatment is utilized to classify the TME immunoarchitecture. As can be observed, the metric change of less than 1% is related to cold TME patterns, while metric change between 1% to 10% is associated with the mixed structures. Finally, the metric change above 10% happens when the compartmentalized pattern is observed at the end of treatment. Moreover, the Shannon’s entropy change (octagon markers) is higher than other metrics for different patterns. All the R cases have compartmentalized TME at the end of the treatment, while NR cases can have either cold or mixed TME. These results illustrate the opportunities but also the challenges in characterizing tumor immunoarchitecture using quantitative metrics and identifying predictive biomarkers.

## 5. Discussion

In the current research, we use and validate our spQSP platform in its ability to quantify intratumoral heterogeneity under anti-PD-1 therapy. Comprehensive quantitative understanding of intratumoral heterogeneity behavior during cancer treatment can lead to matching patients with the best mode of treatment. In the field of cancer immunology, validating the performance of any spatio-temporal algorithm is challenging as there is no standard and systematic approach to quantitatively validate and compare the simulated results with the data derived from spatial biomarkers from digital pathology images. Lack of a sufficient number of digital pathology images obtained from immunohistochemistry (IHC) and immunofluorescence (IF) images can make this task even more challenging. It is worth mentioning that once a spatio-temporal algorithm is quantitively validated, it can be utilized with increased credibility to model TME temporal dynamics, which is not achievable either clinically or experimentally. Here, we use mixing score, average neighbor frequency, Shannon’s entropy and the AUC G-Cross function to quantify intratumoral heterogeneity. These metrics have also been used in different studies to analyze digital pathology images to understand the treatment efficacy. In some of these studies, three immunoarchitectural patterns have been identified within the TME based on the treatment efficacy [3,32]. Cold patterns are referred to the ones with low degrees of immune cell infiltration and are linked to poor treatment response or poor survival. In the mixed patterns, the immune and cancer cells are mixed with no distinct regions mainly occupied by one of them. Mixed structures can be a signature of insufficient treatment. Finally, compartmentalized patterns encompass regions dominantly occupied by either tumor or immune cells. Compartmentalized immune structure can be an indicator of an effective treatment or better survival.

To qualitatively validate the spQSP results, the first two cases are simulated, representing effective and ineffective treatment with respect to the case with no anti-PD-1 therapy (NT). In the simulations, the treatment efficacy is controlled by the administered drug dosage. The results demonstrate that when the drug dose is high (HD), tumor progression is inhibited; meanwhile, in low dose (LD) cases, the treatment cannot stop tumor growth (Figure 2b). In a HD scenario, the TME changes from cold to mixed and finally becomes compartmentalized (Figure 3). However, the LD case morphology remains mixed after conversion from its initial cold pattern (Figure 4). In qualitative agreement with the literature, the compartmentalized immunoarchitectural patterns are observed in the case with better treatment. To quantitatively verify the spQSP-simulated results, the mentioned spatial metrics are computed over time on a fixed 2-D ROI as shown in Figure 2a. In addition to the validation, the other aim of this study is to investigate if the introduced metrics can reflect the change in TME morphology for both effective and ineffective immune checkpoint inhibitor treatments. This can help to select proper metrics to track the intratumoral heterogeneity temporal behavior and link it to TME patterns and treatment outcome. Finally, a bivariate classification based on the ratio of cancer cells to immune cells is proposed to investigate how the ROI with the same ratio of cancer cells to immune cells may have different values of spatial metrics. To more accurately validate spQSP and understand the behavior of each spatial metric, 10 cases, including 5 R cases and 5 NR cases, are simulated and the percent change in each spatial metrics and cancer cell to immune cell ratio are calculated BT and AT.

The spQSP results indicate that the following correspond to compartmentalized immunoarchitecture and better treatment: lower mixing score; lower average neighbor frequency; higher Shannon’s entropy; and lower AUC of the G-cross function, in agreement with the literature. Among the metrics, the change in average neighbor frequency starts earlier than other metrics. However, the bivariate classification based Shannon’s entropy and the cancer cell to immune cell ratio can better reflect the difference between different TME patterns. Mixing score and the AUC of the G-cross function cannot always be used to classify the TME structure because they are not sensitive enough to the change in TME morphology. The change in the TME patterns and spatial metrics are more significant when the tumor vascular density increases and when the treatment has a higher impact on tumor growth inhibition (Figure 9 and Figure 10). The behavior of the spatial metrics in R and NR cases reveals that the percent change in the metrics in R cases is higher than in NR cases. The change in average neighbor frequency is higher than mixing score and the AUC of the G-cross function (Table 1). Moreover, Shannon’s entropy is more sensitive to the ratio of cancer cells to immune cells. The spQSP platform is a 3-D hybrid platform composed of the QSP model and the ABM. Compared to existing spatio-temporal algorithms to simulate tumor evolution, spQSP has potential to generate more realistic results due to its combination with whole-patient dynamics from QSP with respect to a stand-alone ABM. We believe that the proposed approach to quantitively validate the spQSP results can be the first step towards using and modifying this platform for more specific data from digital pathology images to select the best mode of action for cancer treatment. Direct head-to-head comparison and quantitative agreement with real data would be beneficial. It is worth mentioning that the main purpose of this paper is to validate the spQSP model and explore the performance of four metrics of intratumor heterogeneity under different scenarios in a more comprehensive manner. We plan to conduct such comparisons in our future work. However, intratumoral heterogeneity is a complex phenomenon that can involve multiple cell types, genetic and epigenetic alterations and environmental factors. It is impossible to capture the full complexity of these phenomena in ABMs, and simplifying assumptions are required that may affect the accuracy of the model. For example, the number of cell types and cytokines are larger than what has been included in this version of spQSP [3,35,46]. Other cell types (macrophages, helper T cell, Myeloid-derived suppressor cells) and cytokines (CCL2, NO, Arg-I) can readily be added to the existing setup [18,19] but it increases computational time. One novel way to decrease the computational time is to use machine-learning algorithms to generate either more intelligent agents or a faster surrogate model to duplicate the behavior observed in the immunopathological multiplexed samples [54]. Finally, we acknowledge that there are many tunable features of the model, and the variants presented in this paper are just a starting point. In future work, we plan to customize the model based on available data and expand the validation to other tumors and treatment scenarios to provide a more comprehensive sensitivity analysis.

## 6. Conclusions

To characterize intratumoral heterogeneity within the tumors simulated using our previously developed spQSP platform, we utilized four spatial metrics, including mixing score, average neighbor frequency, Shannon’s entropy and the AUC of the G-cross function, together with the ratio of cancer cell number to immune cell number. The relative values of all the time-averaged metrics for high dose (HD) and low dose (LD) immune checkpoint inhibitors are in qualitative agreement with the literature. The qualitative agreement of the metrics can be considered as a novel way to validate the performance of spQSP. Among these metrics, average neighbor frequency and Shannon’s entropy, along with the ratio of cancer cells to immune cells, can be used to classify immunoarchitecture as “cold”, “compartmentalized” and “mixed”. The spQSP results reveal that the compartmentalized structure is observed in the case with a better treatment effect. In addition, the simulations show that mixing score and the AUC of the G-cross function are not as suitable as the other two metrics for TME classification. This sort of TME classification can be used for different ranges of cancer cells and immune cells. The value of Shannon’s entropy can change more significantly as the TME morphology changes. This behavior makes this metric a more suitable candidate for classifying the TME structure based on the treatment outcome. These conclusions are further corroborated by our simulations of groups of responders (R) vs. non-responders (NR). They show the small percentage changes in the metrics in the case of “cold” and “mixed” patterns in the NR group and larger percentage changes in “compartmentalized” patterns corresponding to the R group. In contrast to clinical and experimental results, the validated spQSP allows us to observe, in silico, the temporal behaviors of various biomarkers by visualization and the measurement of intratumoral heterogeneity. The metrics can be used to reflect TME immunoarchitecture, which appears to be predictive of the treatment efficacy. This methodological study provides a foundation for more detailed and concrete studies based on specific cancer types, drugs as single or combination therapies and immunopathological multiplexed samples. 

## Figures and Tables

**Table 1:** Cancer to immune cell ratio and the change percentage of different spatial metrics within the ROI for R and NR cases. BT = before treatment; AT = after treatment. The percent changes in spatial metrics in R cases are higher than those in NR cases. In spite of Shannon’s entropy change, mixing score, average neighbor frequency and the AUC of the G-cross change for R cases are higher when the ratio of cancer cells to immune cells is lower. Change in Shannon’s entropy strongly depends on the cancer cell to immune cell ratio.

	Cell Ratio			Mixing Score	Average Neighbor Frequency	AUC of G-Cross Function	Shannon’s Entropy
	BT	AT	Reduction (%)	Reduction (%)	Reduction (%)	Reduction (%)	Increase (%)
R1	2.86	0.43	84.96	64.03	67.25	34.71	310.13
R2	15.41	5.71	67.94	47.98	49.87	14.53	421.13
R3	3.67	0.57	84.46	63.35	64.92	30.81	300.76
R4	17.36	6.91	65.19	44.15	46.26	12.06	433.78
R5	3.45	0.49	85.79	64.69	67.38	34.67	347.19
NR1	3.19	2.84	10.97	2.82	3.28	2.91	4.24
NR2	16.92	13.46	20.45	0.78	1.71	0.81	2.31
NR3	19.64	15.41	21.53	0.0	0.86	0.0	0.93
NR4	6.94	5.81	16.28	2.68	2.87	1.71	3.92
NR5	18.49	14.56	21.25	0.68	1.21	0.67	2.15

## Data Availability

The authors confirm that the data supporting the findings of this study are available within the article. C++ code, input parameters and the details of the QSP model are public at https://github.com/popellab/spQSP_ITH (accessed on 12 May 2023).
